# Mixed Medicinal Mushroom Mycelia Attenuates Alzheimer’s Disease Pathologies In Vitro and In Vivo

**DOI:** 10.3390/cimb45080428

**Published:** 2023-08-15

**Authors:** Ji Heun Jeong, Geum-Lan Hong, Young Gil Jeong, Nam Seob Lee, Do Kyung Kim, Jong Yea Park, Mina Park, Hyun Min Kim, Ya El Kim, Yung Choon Yoo, Seung Yun Han

**Affiliations:** 1 Armed Forces Medical Research Institute (AFMRI), Daejeon 34059, Republic of Korea; jiheun4110@gmail.com; 2 Department of Anatomy, College of Medicine, Konyang University, Daejeon 35365, Republic of Korea; ghdrmafks@o.cnu.ac.kr (G.-L.H.); ygjeong@konyang.ac.kr (Y.G.J.); nslee@konyang.ac.kr (N.S.L.); dokyung@konyang.ac.kr (D.K.K.); 3 Giunchan Co., Ltd., Cheonan 31035, Republic of Korea; agnes223@daum.net (J.Y.P.); guc2103@guc.co.kr (M.P.); guc2104@guc.co.kr (H.M.K.); guc2105@guc.co.kr (Y.E.K.); 4 Department of Microbiology, College of Medicine, Konyang University, Daejeon 35365, Republic of Korea; yc_yoo@konyang.ac.kr

**Keywords:** Alzheimer’s disease, medicinal mushroom, amyloid β, zinc, 5XFAD

## Abstract

Alzheimer’s disease (AD) is characterized by memory impairment and existence of amyloid-β (Aβ) plaques and neuroinflammation. Due to the pivotal role of oxidative damage in AD, natural antioxidative agents, such as polyphenol-rich fungi, have garnered scientific scrutiny. Here, the aqueous extract of mixed medicinal mushroom mycelia (MMMM)—*Phellinus linteus*, *Ganoderma lucidum*, and *Inonotus obliquus*—cultivated on a barley medium was assessed for its anti-AD effects. Neuron-like PC12 cells, which were subjected to Zn^2+^, an Aβ aggregator, were employed as an in vitro AD model. The cells pretreated with or without MMMM were assayed for Aβ immunofluorescence, cell viability, reactive oxygen species (ROS), apoptosis, and antioxidant enzyme activity. Then, 5XFAD mice were administered with 30 mg/kg/day MMMM for 8 weeks and underwent memory function tests and histologic analyses. In vitro results demonstrated that the cells pretreated with MMMM exhibited attenuation in Aβ immunofluorescence, ROS accumulation, and apoptosis, and incrementation in cell viability and antioxidant enzyme activity. In vivo results revealed that 5XFAD mice administered with MMMM showed attenuation in memory impairment and histologic deterioration such as Aβ plaque accumulation and neuroinflammation. MMMM might mitigate AD-associated memory impairment and cerebral pathologies, including Aβ plaque accumulation and neuroinflammation, by impeding Aβ-induced neurotoxicity.

## 1. Introduction

Alzheimer’s disease (AD) is a chronic neurodegenerative ailment that leads to dementia and is responsible for 75% of all cases of cognitive decline [1]. The principal symptoms associated with AD encompass cognitive impairment and memory deficit [2]. The present pharmacological treatment for AD involves acetylcholinesterase inhibitors, which aim to alleviate these symptoms, but their efficacy is limited as they fail to address the underlying causes of AD [3]. Although the etiology of AD is not yet fully comprehended, it is well established that the extracellular deposition of amyloid-beta (Aβ) protein aggregates represents a crucial hallmark of AD pathology [4]. The aggregated Aβ generates neurotoxic species, which deposit in the brain and give rise to neuroinflammation, neurite degeneration, and neuronal demise, ultimately leading to cognitive decline [5].

Mechanistically, the detrimental effects of Aβ-induced neurotoxicity are widely recognized to arise from disrupted synaptic calcium ion (Ca^2+^) handling resulting from excessive stimulation of glutamate receptors, specifically, N-methyl-D-aspartate receptors (NMDARs) [6]. Numerous studies have previously reported potential associations between NMDARs and oligomeric Aβ. Firstly, NMDAR function may constitute a critical downstream target of Aβ [7]. Secondly, NMDARs may be indispensable for Aβ’s effects on synaptic transmission and plasticity [8]. Thirdly, NMDAR may serve as a receptor for Aβ, either directly or indirectly [9,10]. Notwithstanding the aforementioned, the hyperactivation of calcium-permeable NMDA receptors gives rise to an elevation in intracellular calcium concentration ([Ca^2+^]*_i_*), culminating in the accumulation of reactive oxygen species (ROS) and proteolytic degradation, activated by calpain, which is rendered active by increases in [Ca^2+^]*_i_* [11,12]. This culminates in a pernicious cycle of apoptotic cell death [13].

In addition to the sequence of events pertaining to NMDAR and its downstream pathways, neuroinflammation, aggravated by activated microglia and astrocytes, either via neuronal apoptotic debris or Aβ itself, is also implicated in the pathogenesis of AD [14]. At the onset of AD, both neuronal apoptosis and neuroinflammation contribute to a time-dependent propagation of neuronal damage from the initial foci to the contiguous regions [15]. Thus, it is imperative to develop efficacious pharmacotherapies that specifically target the deleterious events associated with Aβ aggregation, Aβ-induced neurotoxicity, and neuroinflammation.

Mushrooms, among various natural organisms, have not garnered the same reverence as plants, yet numerous studies conducted over the past decades have addressed a wide array of biological activities. Mushrooms represent significant natural resources in human existence, not solely due to their distinctive flavor but also as a source of health-promoting compounds that exhibit antioxidant, antibacterial, anti-inflammatory, and anticancer effects [16,17,18,19]. Among these mushrooms, *Phellinus linteus* (PL), *Ganoderma lucidum* (GL), and *Inonotus obliquus* (IO), renowned as “medicinal mushrooms” in Asian countries, are increasingly attracting scientific attention [20,21,22]. Recent studies, conducted either in vivo or in vitro, have demonstrated that all of the aforementioned species possess the potential to counteract the progression of AD. Remarkably, it has recently come to light that IO can impede Aβ aggregation through hispidin derivatives, which are extracted from IO [23]. Furthermore, it has been established that PL and GL also possess the capacity to inhibit Aβ aggregation by exerting chelating activity against heavy metals such as Zn^2+^ and Cu^2+^, both known to induce Aβ aggregation [24,25].

Based on the available evidence, it is conceivable that a combination of PL, GL, and IO may achieve therapeutic synergy in AD. Nevertheless, the paucity of scientific studies on this matter remains evident, as co-cultivation has proven to be challenging. Nonetheless, with the advent of modern solid-state fermentation (SSF) technology, the cultivation of multiple mushrooms as mycelia in a cost-effective manner, utilizing various types of grains as growth media, has become feasible [26].

In a previous study, we reported that the aqueous extract of a mixed medicinal mushroom mycelia (MMMM; PL, GL, and IO) cultivated via the SSF method demonstrated a protective effect against experimental focal cerebral ischemia [27]. Here, to explore the potential modulatory effect of the water extract of MMMM on AD, we employed an in vitro AD model in which the neuron-like PC12 cell line was exposed to Zn^2+^, an intracellular Aβ accumulation inducer, in accordance with a previous report [28]. Furthermore, we employed 5XFAD transgenic mice as an in vivo AD model, as they faithfully replicate the amyloidopathy observed in human AD.

## 2. Materials and Methods

### 2.1. Preparation of Aqueous Extract of MMMM

The aqueous extract of MMMM was obtained and treated by Giunchan Co., Ltd., situated in Cheonan, Republic of Korea. Specifically, mycelia of PL, GL, and IO, procured from the Korean Forest Research Institute (located in Wanju, Republic of Korea), were introduced onto potato dextrose agar plates (Acumedia, Lansing, MI, USA) and incubated at a temperature of 25 °C for a duration of 7 days. The ensuing mycelial disc, measuring 8 mm in diameter, was divided into 5–6 discs using a sterilized corn borer. Subsequently, the discs were transferred to potato dextrose broth (Acumedia) and continuously stirred at 25 °C for a period of 7 days. After incubation, the mycelia were blended in a 400 Mark Ⅱ stomacher (Seward Laboratory Systems Inc., Port St. Lucie, FL, USA) and transferred to barley flour, which had been soaked in distilled water for 60 min and sterilized thereafter. This concoction was then incubated at a temperature of 25 °C for a span of 10 days. The resulting infused barley flour, containing the three mycelia, was subsequently soaked in distilled water for 60 min and sterilized via autoclaving at a temperature of 121 °C for a duration of 15 min. The aqueous essence of MMMM was then derived through a process of stationary maceration with ethanol for a period of 48 h (using a ratio of 1:2 *w*/*v*), following which the residue was extracted with 70% ethanol for a duration of 24 h (using a ratio of 1:1 *w*/*v*). The extracts were combined, and the solvent was eliminated through reduced pressure at a temperature not exceeding 45 °C, resulting in a desiccated crude extract of MMMM (with an extractive yield of 5.07%). Since our previous study has indicated that the aqueous portion of MMMM demonstrates the least toxicity on PC12 cells [27], the aqueous extract (hereinafter simply referred to as MMMM) was prepared using the fractionation techniques outlined elsewhere, resulting in an extractive yield of 3.93%. The outcomes derived from the chemical characterization of MMMM, which were attained through UHPLC–MS (ultra-high performance liquid chromatography–tandem mass spectrometry) and cross-referenced with the NIST23 Agilent mass spectral library, are accessible in our antecedent investigation [27]. 

### 2.2. Cell Culture

The PC12 cells, a neuron-like cell line originating from a rat pheochromocytoma, were procured from the esteemed Korean Cell Line Bank (Seoul, Republic of Korea). The cultivation of this cell was executed in a medium that consisted of Dulbecco’s modified Eagle’s formulation, which was enriched with a 10% (*v*/*v*) concentration of fetal bovine serum and a 1% penicillin/streptomycin mixture. This cultivation process took place in an environment that was appropriately humidified, containing a 5% CO_2_ content, maintained at a temperature of 37 °C. All the above products were obtained from HyClone (Logan, UT, USA).

### 2.3. Cell Viability

The PC12 cells were seeded at a density of 1 × 10^4^ cells/well in a 96-well plate and incubated overnight to stabilize. To presume the median lethal dose (LD_50_) value of Zn^2+^ and the maximum safe dose of the MMMM, PC12 cells were incubated with various concentrations of ZnCl_2_ (0–150 mM) or the MMMM (0–100 μg/mL) diluted in the medium for 3 and 16 h, respectively. After obtaining the LD_50_ of 3 h of incubation with Zn^2+^ in the form of ZnCl_2_, which was diluted in the medium and allowed to incubate at 37 °C, as 50 μM, the cells were pretreated with 10 μg/mL (MMMM-L) or 100 μg/mL (MMMM-H) for 16 h (hereinafter refer**r**ed to as MMMM-L or MMMM-H groups, respectively) and further incubated with 50 μM Zn^2+^ for 3 h. The cells incubated with only 50 μM Zn^2+^ for 3 h were labeled as Zn^2+^ group. The cells that did not receive either MMMM or Zn^2+^ were designated as the control (Cont) group. Then, the (4,5-dimethylthiazol-2-yl)-2,5-diphenyl tetrazolium bromide (MTT) solution (Sigma-Aldrich, St. Louis, MO, USA) was added to each well at a final concentration of 0.5 mg/mL and incubated for 4 h at 37 °C. The formed formazan crystals were dissolved in 100 μL DMSO after removing the supernatants. Then, the absorbance was determined at 570 nm by using an Lx800 UV microplate reader (Bio-tek Instruments, Winooski, VT, USA). Data were expressed as a percentage of the control.

### 2.4. Immunofluorescence

The PC12 cells were seeded at a density of 1 × 10^5^ cells per well in a 12-well plate, on which the sterilized 15 Ø coverslip was positioned at the base. Following the preparation of different cell groups in accordance with the identical schedule described above, the cells were fixed in 4% paraformaldehyde (PFA) for 60 min. Subsequently, the cells were permeabilized with 0.1% Triton X-100, diluted in phosphate-buffered saline (PBS) at 22 °C for 5 min, and then obstructed in 1% bovine serum albumin diluted in PBS for 60 min at 22 °C. The cells were then incubated with mouse anti-Aβ oligomer (1:500; Abcam, Cambridge, UK), which specifically detects the toxic form (oligomer; aggregated) rather than the non-toxic form (monomer; non-aggregated) of Aβ, which was used as the primary antibody for 24 h. After washing with PBS, the cells were incubated with Cy2-conjugated anti-mouse IgG (Jackson, West Grove, PA, USA), which was used as a secondary antibody for 2 h and counter-stained with 4′,6-diamidino-2-phenylindole (DAPI). The resulting fluorescence was detected by confocal laser scanning microscopy (LSM900, Zeiss, Oberkochen, Germany). The mean intensity per single cell was analyzed with ImageJ (v1.49, National Institutes of Health, Bethesda, MD, USA) and expressed by folds of control.

### 2.5. TUNEL Assay

Apoptosis was measured using the terminal deoxynucleotidyl transferase dUTP nick-end labeling (TUNEL) assay kit (Promega, Madison, WI, USA). In brief, PC12 cells were seeded at a density of 1 × 10^5^ cells/well in a 12-well plate, for which the sterilized 15 Ø coverslip was located at the bottom. Following the preparation of different cell groups in accordance with the identical schedule described above, the cells were treated with the TUNEL reaction mixture and incubated for 60 min at 37 °C in a humid chamber. The cells were briefly washed with PBS and stained with Hoechst 33258 (Sigma-Aldrich) for 15 min at 22 °C to visualize nuclei. The cells were mounted with an anti-fade mounting medium (Fluoroshield^®^, Sigma-Aldrich) and visualized using LSM900 confocal laser scanning microscopy (Zeiss). We randomly measured 45 cells from each experiment to calculate the apoptotic rate. The experiments and the data acquisition were carried out in triplicate.

### 2.6. Flow Cytometry

To quantify PC12 cell apoptosis in response to the different treatments, Muse^TM^ Cell Analyzer (Merck-Millipore, Burlington, MA, USA), an automated fluorescence-activated cell sorting (FACS) device, and the corresponding fluorescein isothiocyanate (FITC) conjugated to Annexin V/7-Aminoactinomycin D (7-AAD) Apoptosis Detection Kit (Merck-Millipore) were used. The Kit utilizes an FITC conjugated to Annexin V as an apoptotic cell marker and 7-AAD as a dead cell marker. For this, the PC12 cells were seeded at a density of 1 × 10^5^ cells/well in a 12-well plate. Following the preparation of different cell groups in accordance with the identical schedule described above, the cells underwent FACS procedures according to manufacturer’s provided protocols. The data and scatter plots were automatically obtained from software embedded in the device. The experiments and the data acquisition were performed in triplicate.

### 2.7. Intracellular ROS Detection 

Measurement of the intracellular ROS level was performed using a 2′-7′-Dichlorodihydrofluorescein diacetate (H_2_DCFDA) (Sigma-Aldrich), an ROS probe. In brief, the cells were incubated with 100 μM H_2_DCFDA diluted in the medium for 30 min at 37 °C. After discarding the excess probe using PBS wash, the cells were further incubated with or without 50 μM Zn^2+^, diluted in the medium for 10 min. At the end of the incubation, the resulting fluorescence was detected with a fluorescence live cell movie analyzer (JuLI-FL^®^, Pleasanton, CA, USA) at excitation/emission wavelengths of 395/509 nm. The experiments and the data acquisition were carried out in triplicate.

### 2.8. Superoxide Dismutase (SOD) and Catalase (CAT) Activity Assay

The activity of SOD and CAT was measured using the corresponding commercial kits based on enzyme-linked immunosorbent assay (ELISA) (BIOMAX, Seoul, Republic of Korea). The PC12 cells were seeded at a density of 1 × 10^6^ cells/well in a 6-well plate. Following the preparation of different cell groups in accordance with the identical schedule described above, the cells underwent ELISA assay according to the protocols provided by the manufacturer. The SOD or CAT activity was determined by a colorimetric method and the absorbance was read at 450 nm or 570 nm, respectively, using an Lx800 UV microplate reader. The experiments and the data acquisition were performed in triplicate. 

### 2.9. Animals and MMMM Administration

The 5XFAD (B6SJL-Tg (APPSwFlLon, PSEN1*M146L*L286V)6799 Vas/J) mice were described by Oakley et al. [29] and obtained from Jackson Laboratory (Bar Harbor, ME, USA, stock number 006554). These mice overexpress both mutant human APP (695) with the Swedish (K670N, M671L), Florida (I716V), and London (V717I) Familial AD mutations, as well as human PS1 harboring two mutations, M146L and L286V. Expression of both transgenes is regulated by neural-specific elements of the mouse Thy1 promoter to drive overexpression in the brain. The 5XFAD transgenic male mice were crossed with B6SJLF1/J female mice (Jackson Laboratory, stock number 100012). The resulting F2-offspring were used in all experiments. Transgenic mice were identified by PCR according to the supplier’s protocol. The mice were kept in conditions at controlled temperature (22 °C) and humidity (40–60%) under 12:12 h light/dark cycles with access to food and water ad libitum. All animal experiments were conducted in accordance with the National Institutes of Health Guidelines for the Care and Use of Laboratory Animals [30] and the guidelines of the ethical procedures and scientific care by the Institutional Animal Care and Use Committee at Konyang University (Approval number: P-21-14-A-01). The transgenic mice (males, 4 months old) were randomly divided into two groups (*n* = 5 each): the “Tg” group, consisting of transgenic mice administered tap water as a vehicle for future use, and the “Tg + MMMM” group, consisting of transgenic mice administered MMMM in the future. The group of wild-type littermates with the same sex and age (*n* = 5), referred to as the “WT” group, was given tap water. For the Tg + MMMM group, MMMM was diluted in 1 mL of tap water and orally administered daily, resulting in a final concentration of 30 mg/kg/day for 8 weeks. The concentration was referenced from the concentration used in our previous report [27]. For the Tg and WT groups, 1 mL of tap water was administered using the same route and duration. All three groups underwent two separate behavioral tests: the Y-maze test (Y-MT) and the passive avoidance test (PAT), after which the animals were sacrificed for tissue sampling. The chronological sequence of in vivo experiments is illustrated in the Figure in Section 3.4.

### 2.10. Y-Maze Test (Y-MT)

The Y-MT was performed to assess spatial learning and memory. Each mouse was placed into the center of the black plastic maze with three arms (30 cm in length, 8 cm in width, and 15 cm in height) at 120-degree intervals. The sequence and number of arm entries were monitored for a 5 min period. To calculate the percentage of spontaneous alternation, the following formula was used: % spontaneous alternation = [(Number of alternations)/(Total arm entries − 2)] × 100. The number of arm entries was used as an indicator of locomotor activity. The overall experimental conditions were conducted in a dark environment, and the maze was cleaned with 70% alcohol at the end of each test. 

### 2.11. Passive Avoidance Test (PAT) 

The PAT was employed as the second tool to assess the memory performance. The PAT apparatus was equipped with 2 juxtaposed illuminated and dark chambers, each 20 × 20 × 20 cm in size. A 50 W lamp was placed in the illuminated chamber. Each test involved 2 separate sessions: a training session and a test session. During the training session, the mouse was initially placed in the illuminated chamber. When the mouse entered the dark chamber, the “initial” latency period was measured using a stopwatch. Subsequently, the mouse received an electrical shock (0.5 mA, delivered through stainless steel rods) for 3 s in the dark chamber. A test session was performed 24 h after the training session. In this session, the mice were located in the illuminated chamber and their “step-through” latency to re-enter the dark chamber was measured (up to 60 s). After each rat was tested, the walls of the chambers were cleaned using 70% ethanol.

### 2.12. Brain Tissue Preparation

After all behavioral tests were finished, the mice were anesthetized with 2% isoflurane in 70% N_2_O and 30% O_2_ under spontaneous respiration and transcardially perfused with 4% PFA. The brain was quickly isolated, post-fixed with 4% PFA overnight at 4 °C, dehydrated with a graded ethanol series, embedded in paraffin, and serially sectioned at 5 μm thickness using a microtome (RM2255; Leica, Wetzlar, Germany). All sections were mounted on 0.01% Poly-D-L-lysine-coated glass slides. Among them, only the hippocampus-bearing slides were selected and the tissues collected from each individual were stored at 22 °C until their use.

### 2.13. Thioflavin S Staining

Two slides were randomly selected from the hippocampus-bearing tissue collections of each mouse (*n* = 5 each), deparaffinized in xylene, hydrated by a decreasing ethanol gradient series, and washed twice in distilled water. The sections were incubated with 1% thioflavin S (Thio-S; Sigma-Aldrich), a specific staining tool for the amyloid β plaques, and dissolved in 50% ethanol at 22 °C for 10 min. Coverslips were mounted on slides and observed under an LSM900 confocal laser scanning microscopy (Zeiss). Under a fluorescence illumination setting (excitation/emission wavelength: 391/428 nm), both hippocampi were photographed and the total fluorescence areas in a high-power field (HPF, X200) were averaged. 

### 2.14. Immunohistochemistry

Two slides were randomly selected from the hippocampus-bearing tissue collections of each mouse (*n* of each group = 5), deparaffinized in xylene, hydrated by a decreasing ethanol gradient series, and washed twice in distilled water. After quenching by endogenous peroxidase with 1% H_2_O_2_ for 1 h at 22 °C, the sections were incubated with primary antibodies: mouse anti-glial fibrillary acidic protein (GFAP; Invitrogen, Rockford, IL, USA) and rabbit anti-Iba-1 (Wako, Richmond, VA, USA) antibodies, both of which were diluted in PBS at a ratio of 1:200 in a humid chamber for 24 h at 4 °C. After washing with PBS, the sections were incubated with secondary antibodies: biotinylated anti-mouse or anti-rabbit IgG antibodies (Vector laboratories, Burlingame, CA, USA), respectively, which were diluted in PBS at a ratio of 1:250 in a humid chamber for 2 h at 22 °C. After washing with PBS, the sections were further incubated with an avidin–biotin complex (VECTASTAIN ABC kit; Vector) for 1 h at 22 °C. The positive reactions were visualized with 3,3′-diaminobenzidine tetrahydrochloride (DAB, Vector). After mounting, the hippocampi of each section were photographed by light microscope (DM4; Leica). The area of GFAP- and Iba-1-immunoreactive (^+^) foci in each HPF was quantified using ImageJ. Data were expressed as a percentage of the WT group.

### 2.15. Statistical Analysis

All data are expressed as the mean ± standard error of the mean (SEM). Statistical analysis was performed using a one-way ANOVA, followed by a Tukey post hoc test for pairwise comparisons. Values of *p* < 0.05 were considered statistically significant. All calculations were performed using GraphPad Prism (Graph-Pad Software 6, San Diego, CA, USA).

## 3. Results

### 3.1. MMMM Impedes Aβ Aggregation and Mitigates Associated Cell Death in PC12 Cells

**Figure 1:**
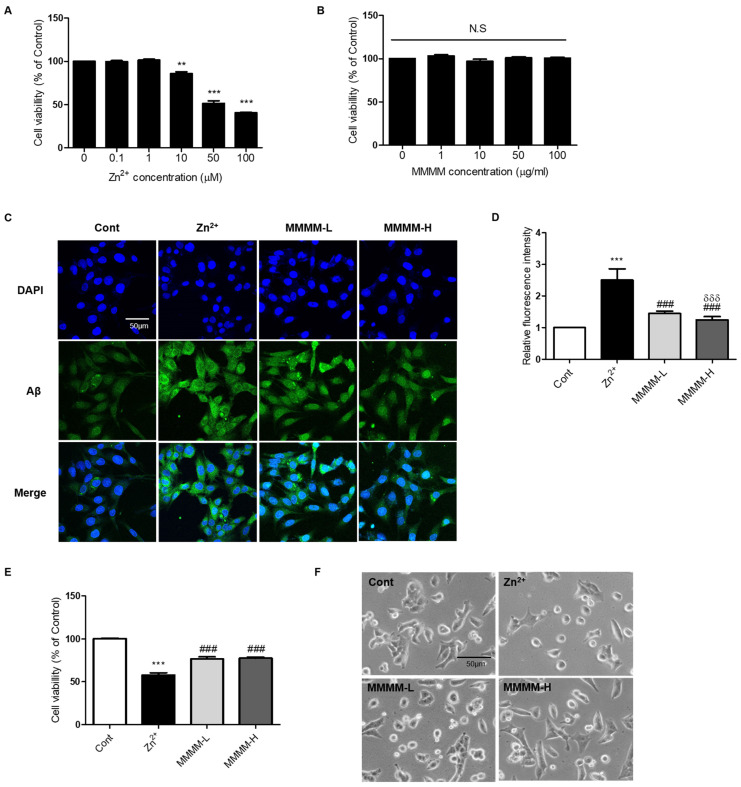
Effects of MMMM on Zn^2+^-induced Aβ aggregation. (**A**) To presume the median lethal dose (LD_50_) value of Zn^2+^, PC12 cells were incubated with various concentrations of ZnCl_2_ (0–100 μM) for 3 h and the resulting cell viability was assessed by MTT assay. (**B**) Bioavailability of the 16 h of incubation with MMMM (0–100 μg/mL) was evaluated in PC12 cells by performing MTT assay. (**C**) Representative immunofluorescence images and (**D**) their quantitative bar graphs showing aggregated Aβ in the cells pretreated with MMMM (10 μg/mL, MMMM-L; 100 μg/mL, MMMM-H) for 16 h and further treated with 50 μM ZnCl_2_ for 3 h. (**E**) Cell viability and (**F**) Morphogloy of cells pretreated with MMMM (10 μg/mL, MMMM-L; 100 μg/mL, MMMM-H) for 16 h and further treated with 50 μM ZnCl_2_ for 3 h). In (**A**,**B**), ** *p* < 0.01 and *** *p* < 0.001, respectively, vs. Cont. In (**D**,**E**), *** *p* < 0.001 vs. Cont; ^###^ *p* < 0.001 vs. Zn^2+^; and ^δδδ^ *p* < 0.001 vs. MMMM-L. Data are represented as the mean ± standard deviation. N.S; no significance.

To establish an in vitro model of AD, we induced Aβ aggregation in PC12 cells by exposing them to Zn^2+^ in the form of ZnCl_2_ [28]. Initially, we determined the LD_50_ values of Zn^2+^ and identified a safe dose of MMMM. PC12 cells were treated with various concentrations of Zn^2+^ (ranging from 0 to 100 μM) and MMMM (ranging from 0 to 100 μg/mL) for 3 h and 16 h, respectively. The LD_50_ value of Zn^2+^ was determined to be 50 μM (Figure 1A), while MMMM was found to be non-toxic at concentrations below 100 μg/mL (Figure 1B). Subsequently, we investigated the effect of Zn^2+^ on Aβ aggregation and the potential attenuation of Aβ aggregation by MMMM, using MMMM-L and MMMM-H groups (refer to the Section 2. The results demonstrated that Zn^2+^ treatment significantly enhanced intracellular aggregated Aβ levels, which were effectively normalized in both the MMMM-L and MMMM-H groups (Figure 1C,D). Furthermore, we examined the potential effects of MMMM on Aβ-induced neurotoxicity (Figure 1E). Viability of cells treated with Zn^2+^ (the Zn^2+^ group) was significantly reduced compared to the non-treated controls (the Cont group), whereas the cell viabilities in the MMMM-L and MMMM-H groups showed remarkable improvements compared to the Zn^2+^ group (77.57 ± 1.64 and 76.90 ± 1.75 vs. 60.32 ± 2.05%, respectively, ^###^ *p* < 0.001). Additionally, we assessed the protective effect of MMMM through morphological observations (Figure 1F). The Zn^2+^ group exhibited cellular shrinkage and shortened cell processes, which are indicative of ongoing neuronal cell death. However, these morphological features were significantly ameliorated in the MMMM-L and MMMM-H groups. These findings suggest that MMMM can inhibit the formation of Aβ aggregation and mitigate Aβ-induced neuronal death.

### 3.2. MMMM Attenuates Aβ-Induced Neuronal Apoptosis

**Figure 2:**
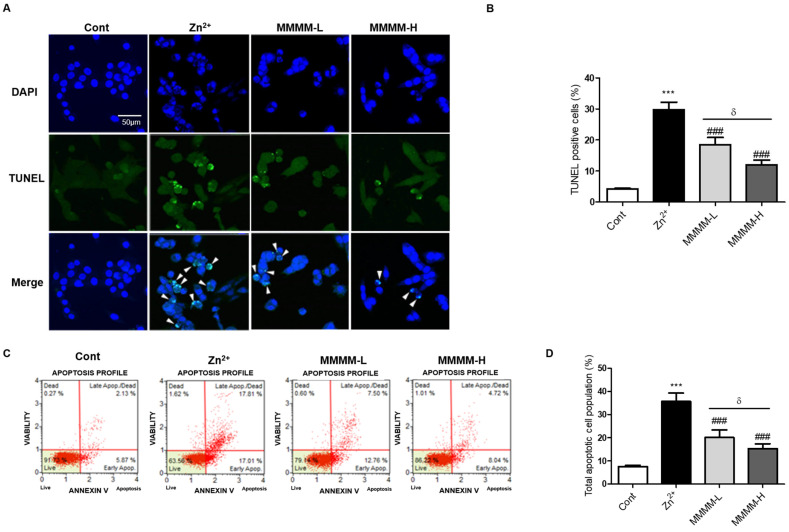
Effects of MMMM on Aβ-induced neuronal apoptosis in in vitro AD models. PC12 cells were pretreated with MMMM (10 μg/mL, MMMM-L; 100 μg/mL, MMMM-H) for 16 h and further treated with 50 μM ZnCl_2_ for 3 h. Using the in vitro AD model, the effects of MMMM on dynamics of ongoing apoptosis were evaluated by (**A**) TUNEL assay and (**B**) the following quantification. After cells underwent FACS and the corresponding kits for detecting fluorescein isothiocyanate (FITC) conjugated to Annexin V/7-Aminoactinomycin D (7-AAD), the resulting (**C**) scatter plots and (**D**) quantitative graphs were obtained. In all graphs, *** *p* < 0.001 vs. Cont; ^###^ *p* < 0.001 vs. Zn^2+^; and ^δ^ *p* < 0.05 vs. MMMM-L. Data are represented as the mean ± standard deviation. TUNEL, a terminal deoxynucleotidyl transferase dUTP nick-end labeling; FACS, an automated fluorescence-activated cell sorting.

To investigate the potential impact of MMMM on apoptosis during the process of Aβ-induced cell death, we conducted TUNEL- and FACS-based apoptosis assays. As depicted in Figure 2A,B, the quantity of TUNEL-positive cells undergoing apoptosis (indicated by a white arrowhead) exhibited a significant increase in the Zn^2+^ group. Conversely, the number of apoptotic cells noticeably decreased in both the MMMM-L and MMMM-H groups. Treatment with either 10 or 100 µg/mL MMMM alone did not exert any discernible influence on the nature of apoptosis in PC12 cells. These findings were corroborated by employing FACS and the Annexin V/7-AAD staining. As illustrated in Figure 2C,D, the Zn^2+^ group displayed a substantial elevation in the overall apoptotic rate when compared to the Cont group (36.06 ± 1.31 vs. 7.80 ± 0.49%, *** *p* < 0.001). However, both the MMMM-L and MMMM-H groups exhibited marked reductions in the total apoptotic rate relative to the Zn^2+^ group (20.11 ± 1.01 and 15.25 ± 0.78%, respectively, ^###^ *p* < 0.001), and these reductions were dose-dependent (^&^ *p* < 0.05). These outcomes suggest that treatment with MMMM can mitigate Aβ-induced apoptosis in neurons.

### 3.3. MMMM Inhibits Aβ-Induced Oxidative Stress in Neuronal Cells 

**Figure 3:**
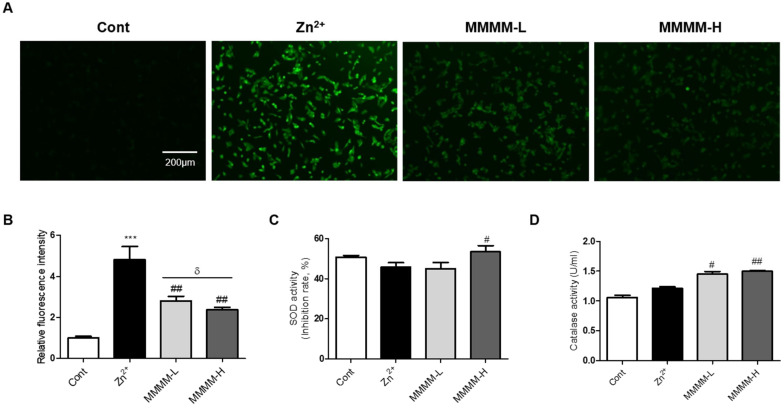
Effects of MMMM on Aβ-induced ROS generation and activities of SOD and Catalase in in vitro AD models. PC12 cells were pretreated with MMMM (10 μg/mL, MMMM-L; 100 μg/mL, MMMM-H) for 16 h and further treated with 50 μM ZnCl_2_ for 3 h. Using the in vitro AD model, the effects of MMMM on intracellular ROS levels was evaluated by (**A**) H_2_DCFDA assay and (**B**) the following quantification of the DCF fluorescence. Activities of (**C**) SOD and (**D**) Catalase were assessed in cells with different treatments. In all graphs, *** *p* < 0.001 vs. Cont; ^#^ *p* < 0.05 and ^##^ *p* < 0.01 vs. Zn^2+^; and ^δ^ *p* < 0.05 vs. MMMM-L. Data are represented as the mean ± standard deviation. ROS, reactive oxygen species; H_2_DCFDA, a 2′-7′-Dichlorodihydrofluorescein diacetate.

To investigate the potential inhibitory effects of MMMM on Aβ-induced ROS production in neuron-like PC12 cells, we employed H_2_DCFDA staining. As depicted in Figure 3A,B, the Zn^2+^ group exhibited a substantial increase in ROS levels compared to the Cont group (4.92 ± 0.04-fold, *** *p* < 0.001). In contrast, both the MMMM-L and MMMM-H groups demonstrated a remarkable reversal of this trend (2.87 ± 0.08 and 2.50 ± 0.09-fold of Cont, ^##^ *p* < 0.01). We further investigated the impact of MMMM on the dynamics of SOD and Catalase activities, which are two major antioxidant enzymes associated with the inhibition of ROS generation. As illustrated in Figure 3C,D, the results revealed significantly higher SOD activity in the MMMM-H group, while both the MMMM-L and MMMM-H groups displayed elevated Catalase activity. These findings suggest that MMMM possesses the capability to suppress ROS generation by enhancing SOD and Catalase activities during the process of Aβ-induced neurotoxicity.

### 3.4. MMMM Prevents Memory Impairments in 5XFAD Mice

**Figure 4:**
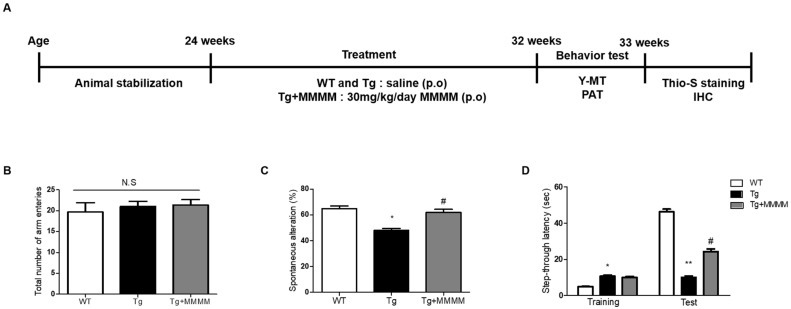
Effects of MMMM on memory impairment in the in vivo AD model. (**A**) The 5XFAD mice were treated with MMMM (30 mg/kg/day) for 8 weeks and underwent the indicated experiments in accordance with desired protocols. Using Y-MT, the extents of (**B**) total arm entries and (**C**) spontaneous alteration in mice groups administered with different treatments were obtained. Using PAT, (**D**) step-through latencies of mice groups administered with different treatments were quantified. In all graphs, * *p* < 0.05 and ** *p* < 0.01 vs. WT; ^#^ *p* < 0.05 vs. Tg. Data are represented as the mean ± standard deviation. p.o, per oral; Y-MT, Y-maze test; PAT, passive avoidance test; Thio-S, Thioflavin S; IHC, immunohistochemistry. N.S; no significance.

To explore the impact of MMMM on memory impairment, a crucial aspect in AD patients, we conducted a Y-MT and PAT using 5XFAD mice, an experimental AD model in vivo, following the designated schedule depicted in Figure 4A. The Y-MT findings indicated no disparity in the total number of arm entries across all groups, indicating that the mutations associated with 5XFAD mice or MMMM treatments did not affect motor function (Figure 4B). Nonetheless, the Tg group displayed a significantly reduced spontaneous alteration value compared to the WT group, demonstrating the presence of memory impairment in the Tg group (49.94 ± 3.18 vs. 64.25 ± 1.74%, * *p* < 0.05; Figure 4C). Interestingly, the Tg + MMMM group exhibited a noteworthy increase in this value compared to the Tg group (62.65 ± 1.75%, ^#^ *p* < 0.05). Furthermore, PAT was employed as another tool to assess memory function (Figure 4D). During the training session, the Tg group exhibited a prolonged step-through latency compared to the WT group, while there was no difference in this value between the Tg and Tg + MMMM groups. During the test session, the Tg group displayed a significant reduction in step-through latency compared to the WT group, indicating the successful induction of memory deficit in the Tg group (7.00 ± 1.25 vs. 45.50 ± 3.63 s, ** *p* < 0.01). Importantly, the Tg + MMMM group demonstrated a significant elongation in step-through latency compared to the Tg group (24.75 ± 4.25 s, ^#^ *p* < 0.05). These results suggest that the intake of MMMM can ameliorate the memory deficits observed in the AD model in vivo.

### 3.5. MMMM Inhibits Aβ Accumulation, Plaque Formation, and Neuroinflammation in the Hippocampus of 5XFAD Mice

**Figure 5:**
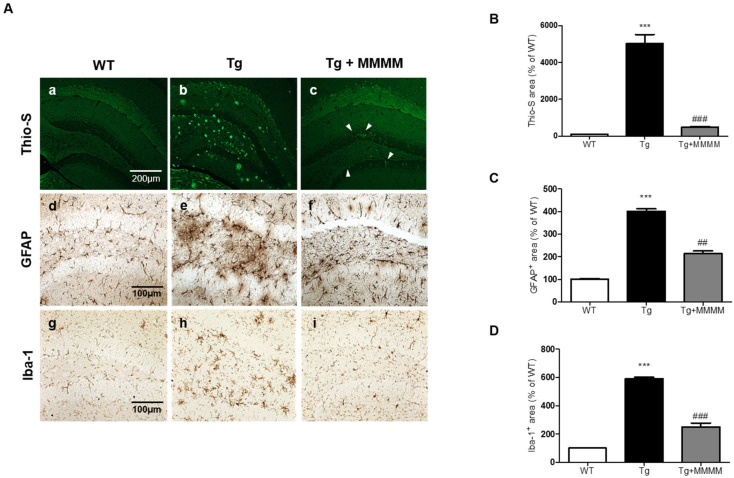
Effects of MMMM on Aβ deposits and neuroinflammation in in vivo AD model. (**A**) Representative images of Thio-S fluorescence (**a**–**c**) and the IHC showing GFAP^+^ astrocytes (**d**–**f**) and Iba-1^+^ microglia (**g**–**i**) in different mice groups. (**B**) Thio-S-stained and (**C**) GFAP- and (**D**) Iba-1-immunostained areas were expressed using quantitative bar graphs. In all graphs, *** *p* < 0.001 vs. WT; ^##^ *p* < 0.01 and ^###^ *p* < 0.001 vs. Tg. Data are represented as the mean ± standard deviation. Thio-S, Thioflavin S; GFAP, glial fibrillary acidic protein; Iba-1, ionized calcium-binding adapter molecule1.

To investigate the potential impact of MMMM on Aβ accumulation in vivo, we conducted Thio-S staining to quantify the level of aggregated Aβ in the hippocampus, utilizing 5XFAD mice. As shown in Figure 5A(a–c),B, the results revealed that the aggregated Aβ was scarcely detectable in the hippocampi of the WT group. Conversely, the Tg group exhibited a substantial increase in the amount of aggregated Aβ, presenting a widely distributed plaque-like appearance. Remarkably, the increment was significantly diminished in the Tg + MMMM group compared to the Tg group. The Thio-S-stained area, representing the region containing aggregated Aβ, was predominantly situated within the neuronal cell bodies (indicated by a white arrowhead in Figure 5A(c)), rather than being present in the extracellular space. These results clearly demonstrate that the administration of MMMM can effectively impede Aβ aggregation, as well as reduce the extracellular accumulation of Aβ plaques in the hippocampus of the AD model in vivo. Next, to investigate whether MMMM affects neuroinflammation, we performed immunohistochemistry using GFAP and Iba-1 antibodies, markers for astrocytes and microglia, respectively. As shown in Figure 5C,D, the densities of both GFAP- and Iba-1^+^ cells were significantly increased in the hippocampi of the Tg group compared with the WT group. Furthermore, these cells observed in the Tg group exhibited mostly ramified morphologies, indicating activated states of each cell type. In contrast, hippocampi of the Tg + MMMM group showed a significant reduction in immunoreactivities of GFAP^+^ astrocytes as well as Iba-1^+^ microglia compared to those of the Tg group. These results suggest that MMMM can suppress neuroinflammation in the hippocampus of the AD model in vivo.

## 4. Discussion

Alzheimer’s disease (AD) constitutes nearly 50–70% of all neurodegenerative disorders related to dementia [31]. With the global aging population, it is predicted that over 130 million individuals aged 65 and above will develop AD by the year 2050 [32]. This disease causes significant social and economic burdens, especially for patients, their families, and caregivers. A hallmark of AD is the accumulation of Aβ plaques, leading to key symptoms such as memory loss, difficulty in recalling recent events, and changes in behavior [33]. Severe cases of AD can result in a loss of communication ability and susceptibility to infections and chronic inflammation, ultimately leading to death [34,35]. Early diagnosis and treatment are crucial, as the disease’s progression leads to irreversible cognitive decline. While there are treatments available for AD, such as donepezil, galantamine, and memantine, their effectiveness is questioned due to the multifactorial and complex nature of the disease [36,37]. Additionally, these medications only provide temporary relief from AD symptoms and have limited efficacy in slowing down the progressive deterioration [38]. More research should focus on the therapeutic management of AD through international interdisciplinary collaboration to address the disease’s mechanisms.

The limitations of current treatments could be addressed by considering the use of complementary and alternative medicines. Highly nutritious culinary and medicinal mushrooms offer abundant sources of antioxidants that have shown promise in delaying the progression of neurodegenerative disorders [39]. Mushrooms contain numerous bioactive compounds, including alkaloids, flavonoids, polyketides, steroids, terpenes, polysaccharides, proteins, micronutrients, and unsaturated fatty acids [40]. Among various mushrooms, PL, GL, and IO have been reported to exhibit outstanding neuroprotective effects [41,42,43]. For instance, PL extract has shown dose-dependent protective effects against oxidative stress-induced apoptosis in human neuroblastoma cells [44]. Similarly, GL has been found to protect cerebellar granule cells against apoptosis induced by H_2_O_2_ [45]. Additionally, IO’s major triterpenoid constituents have demonstrated protective efficacy against H_2_O_2_-induced neurotoxicity in neuron-like neuroblastoma cells [46].

Combining PL, GL, and IO has the potential to synergistically enhance neuroprotective effects, but experimental trials have been limited due to their rarity in nature, methodological challenges, and associated expenses. However, modern SSF-based cultivation techniques have made it possible to produce mixed mushrooms such as PL, GL, and IO in the form of mycelia, enabling experiments to screen for possible neuroprotection [26]. In our previous study, we utilized the water, ethanolic, and ethyl acetate extracts of MMMM to demonstrate their neuroprotective effects in an in vitro ischemic stroke model. Among these extracts, the water extract of MMMM emerged as the most secure and efficacious, unveiling its composition of nine primary isolated compounds, namely Kingianoside B, n-Henicosanal, 6-Gingerol, Vernolic acid, 19-Glucosyl-14-deoxyandrographolide, Matairesinoside, Dibutyl sebacate, 9-Ene-methyl palmitate, and Sanleng acid, impeccably arranged in decreasing order of their retention times through UHPLC–MS analyses [27]. 

Among them, 6-Gingerol, which constitutes a phenolic compound initially isolated from ginger, has been documented to exert antioxidative [47] and anti-inflammatory effects [48]. Notably, in this epoch of neuroprotection, recent evidence has evidenced that 6-Gingerol can mitigate lipopolysaccharide-induced neuroinflammation in vitro and in vivo by suppressing astrocyte hyperactivation [49]. Additionally, 6-Gingerol has been demonstrated to shield against hypoxia-induced apoptosis of PC12 cells through the upregulation of the neuroprotective microRNA-103 [50]. These aforementioned reports have paved the way for the formulation of a hypothesis, positing that the therapeutic mechanisms underlying the anti-AD effect induced by MMMM might involve, at least in part, 6-Gingerol-mediated anti-neuroinflammatory and anti-apoptotic properties. Considering the potential for strategic enhancement of 6-Gingerol through the SSF-based MMMM cultivation method, diverse modifications to culture conditions (e.g., temperature, duration, and mixing ratio of the three mushroom mycelia) may represent a viable and promising strategy.

Despite offering a new perspective, we cannot ignore the fact that there are some limitations to this study, as it solely focused on the therapeutic effect of MMMM without comparing it to PL, GL, or IO used individually in vitro and in vivo. Further detailed comparative analyses with these variants as experimental controls are essential to quantify the actual synergistic effects of the combination. In addition, the study did not assess the possible upstream targets of the antioxidant enzymes upregulated by MMMM, such as the nuclear factor erythroid-derived 2-related factor 2 (Nrf2) [51]. A more detailed analysis of MMMM’s possible modulation of Nrf2/Keap1, the master regulator of anti-oxidative enzymes [52], will be crucial in future investigations. One further limitation of this study is that the precise stage at which the therapeutic effect of MMMM intervention occurs in AD treatment remains unclear. According to several recent studies, intraneuronal Aβ oligomers appear to precede extraneuronal Aβ plaques and are known to induce a significant level of microglia-mediated neuroinflammation, synaptic dysfunction, and neurofibrillary tangle formation in the course of AD pathogenesis [53,54,55]. Building on this, we demonstrated the efficacy of MMMM in the early stage of AD by utilizing the intraneuronal Abeta aggregator, Zn^2+^, in vitro. However, it cannot be ruled out that MMMM may also possess the ability to inhibit neurotoxicity caused by extraneuronal Aβ plaques in the late stages of AD. To address this issue, further in vivo experiments involving mouse groups differentiated by various stages of AD pathogenesis are required.

In conclusion, the results suggest that regular consumption of MMMM may alleviate AD-associated cognitive decline and pivotal pathologies, including Aβ plaque accumulation and neuroinflammation, by impeding intracellular Aβ aggregation and its resulting neurotoxicity. As such, the supplementation of MMMM could be a valuable preventive strategy against AD.

## Data Availability

The datasets generated and analyzed during the current study are available from the corresponding authors on request.
